# Research on a Cooperative Grasping Method for Heterogeneous Objects in Unstructured Scenarios of Mine Conveyor Belts Based on an Improved MATD3

**DOI:** 10.3390/s25226824

**Published:** 2025-11-07

**Authors:** Rui Gao, Mengcong Liu, Jingyi Du, Yifan Bao, Xudong Wu, Jiahui Liu

**Affiliations:** 1 College of Electrical and Control Engineering, Xi’an University of Science and Technology, Xi’an 710054, China; 2 College of Mechanical Engineering, Xi’an University of Science and Technology, Xi’an 710054, China

**Keywords:** cooperative grasping, Multi-Agent Twin Delayed Deep Deterministic Policy Gradient (MATD3), multi-factor reward function, prioritized experience replay (PER), sequence cooperative optimization strategy

## Abstract

Underground coal mine conveying systems operate in unstructured environments. Influenced by geological and operational factors, coal conveyors are frequently contaminated by foreign objects such as coal gangue and anchor bolts. These contaminants disrupt conveying stability and pose challenges to safe mining operations, making their effective removal critical. Given the significant heterogeneity and unpredictability of these objects in shape, size, and orientation, precise manipulation requires dual-arm cooperative control. Traditional control algorithms rely on precise dynamic models and fixed parameters, lacking robustness in such unstructured environments. To address these challenges, this paper proposes a cooperative grasping method tailored for heterogeneous objects in unstructured environments. The MATD3 algorithm is employed to cooperatively perform dual-arm trajectory planning and grasping tasks. A multi-factor reward function is designed to accelerate convergence in continuous action spaces, optimize real-time grasping trajectories for foreign objects, and ensure stable robotic arm positioning. Furthermore, priority experience replay (PER) is integrated into the MATD3 framework to enhance experience utilization and accelerate convergence toward optimal policies. For slender objects, a sequential cooperative optimization strategy is developed to improve the stability and reliability of grasping and placement. Experimental results demonstrate that the P-MATD3 algorithm significantly improves grasping success rates and efficiency in unstructured environments. In single-arm tasks, compared to MATD3 and MADDPG, P-MATD3 increases grasping success rates by 7.1% and 9.94%, respectively, while reducing the number of steps required to reach the pre-grasping point by 11.44% and 12.77%. In dual-arm tasks, success rates increased by 5.58% and 9.84%, respectively, while step counts decreased by 11.6% and 18.92%. Robustness testing under Gaussian noise demonstrated that P-MATD3 maintains high stability even with varying noise intensities. Finally, ablation and comparative experiments comprehensively validated the proposed method’s effectiveness in simulated environments.

## 1. Introduction

Multi-agent cooperative grasping, as a significant research direction in robotics, aims to address practical operational demands in complex tasks such as conveyor belt foreign object sorting, logistics handling, and disaster rescue operations. In such scenarios, multi-agent cooperation can significantly enhance operational efficiency and task success rates. However, current research in this field faces notable limitations: most studies focus on single-agent systems and often assume static, closed-loop, idealized task environments. In the real world, agents must not only continuously interact with their surroundings but also coordinate with other agents. Existing research relies on simplified models that fail to capture the dynamic complexity of multi-agent systems embedded in real-world, open environments [1]. From an application perspective, current foreign object sorting robots utilize artificial intelligence, computer vision, and robotic control technologies to achieve conveyor-based foreign object sorting. Sorting methods include grasping sorting (multi-arm grasping) and pneumatic sorting (high-pressure air sorting devices). Their shared key technologies encompass: intelligent perception of grasping targets, dynamic target-tracking trajectory planning for robotic arms, and intelligent cooperation among multiple robotic arms [2]. Overall, this field is evolving from traditional methods toward data-driven models and expanding from single-agent closed tasks to multi-agent open cooperation.

This paper primarily investigates trajectory planning and intelligent cooperative control for dual-arm systems. Current traditional control algorithms face three core challenges: (1) Limited environmental adaptability: High model complexity struggles to adapt to dynamic operational conditions; (2) Insufficient real-time performance: Excessive computational load causes response delays; (3) Weak task generalization: Inadequate planning capability for heterogeneous grasping targets. For trajectory planning and cooperative grasping algorithms targeting foreign objects on coal mine conveyor belts, traditional approaches fall into three categories: artificial potential field methods (prone to local optima), heuristic algorithms (unstable solution quality), and sampling methods (poor dynamic adaptability). While these methods address cooperative planning in structured scenarios, their heavy reliance on prior global environmental information severely limits generalization in unstructured environments.

Learning-based control methods: These achieve adaptive decision-making through end-to-end learning, hierarchical learning, and imitation learning (representative methods include DQN, DDPG, PPO, BC, IRL and GAIL). By autonomously learning complex high-dimensional state-action mappings, they approximate optimal cooperative strategies, establishing an effective paradigm for dual-arm planning. The following sections will elaborate on the current research status of traditional and learning-based methods.

The artificial potential field (APF) method is a widely applied technique in robotic arm trajectory planning. It constructs gravitational and repulsive fields to avoid obstacles and find optimal paths. However, traditional potential field methods suffer from issues such as local minima and target proximity to obstacles, limiting their application in robotic arm trajectory planning [3,4]. Reference [5] proposes an integrated planning approach based on the APF framework that circumvents the complexity of inverse kinematics while reducing computational load. Reference [6] introduces an improved velocity potential field (IVPF) algorithm, which incorporates directionality, obstacle geometry, and tangential velocity to significantly enhance obstacle avoidance. In [7], a novel 3D path planning algorithm is presented that addresses issues of local minima and trajectory oscillations by employing force sensors to predict obstacle locations, thereby enabling smoother and more stable path generation. While traditional APF-generated paths are intuitive and smooth, these methods are prone to convergence at suboptimal solutions and require sensitive parameter tuning. Reference [8] proposes a dual-arm path planning method using closed-loop constrained kinematics to meet the cooperation constraints of dual manipulators. Reference [9] develops a distributed dual-arm trajectory planning algorithm that employs a tool-centered cooperative system for real-time trajectory correction. For complex tasks such as objects stacking, contact-rich manipulation, or cooperative assembly, more precise motion control is essential. Reference [10] introduces an adaptive gravitational constant and segmented repulsive force model to address common APF drawbacks. The dynamic adjustment of the gravitational constant helps the robot escape local minima, while the segmented repulsive function allows for effective obstacle avoidance even in proximity to obstacles. Reference [11] extends the APF concept to autonomous driving by introducing an adaptive vehicle aggressiveness regulation strategy. This method improves traffic safety and efficiency by optimizing multi-objective criteria—including conflict rates and travel speed—through behavior modulation based on APF principles. Reference [12] further enhances the APF method by bounding Cartesian components of the attractive field and incorporating velocity feedforward control. Theoretical analysis using Lyapunov stability guarantees continuous trajectory following, while a collision detection model establishes repulsive fields between joint-obstacle nearest points, thereby ensuring effective obstacle avoidance for redundant manipulators.

Classic heuristic algorithms used in robotic path planning include A* search [13], Dijkstra’s algorithm, genetic algorithms, particle swarm optimization (PSO), and ant colony optimization (ACO), among others. Reference [14] combines ACO with A* search to develop the ACO-A* algorithm, which is tailored for autonomous underwater vehicles (AUVs) navigating through multiple targets in complex environments with dense obstacles. The ant colony algorithm is employed to sequentially traverse target locations, while A* search is used for fine-grained path planning, demonstrating both the effectiveness and necessity of the proposed method. Reference [15] presents an extended Dijkstra algorithm that utilizes Delaunay triangulation to model the surface environment. This method explores all two-dimensional, unfoldable, and traversable paths to identify the shortest path among all optimal candidates, thereby enhancing surface path accuracy. Reference [16] introduces an Enhanced Genetic Algorithm (EGA) to refine initial trajectories in continuous space, generating an optimal, collision-free path closely aligned with the start and end configurations. Reference [17] proposes a trajectory planning approach for a six-degree-of-freedom (6-DOF) industrial robotic arm based on an Improved Multi-Verse Optimizer (IMVO). By enhancing the wormhole probability distribution, incorporating adaptive parameter tuning, and fusing population mutation strategies, the algorithm improves convergence speed and global exploration capability. This method addresses time-optimal trajectory generation while minimizing energy consumption and mechanical impact. Reference [18] applies the PSO algorithm to 6-DOF robotic arm trajectory planning. A fifth-order polynomial ensures the continuity of position, velocity, and acceleration at the trajectory boundaries. Forward kinematics are employed to compute the joint variables corresponding to tracking points, which are then transformed into Cartesian cooperation to obtain the manipulator’s position. Reference [19] proposes a multi-arm cooperative coal gangue sorting method based on an improved Hungarian algorithm. Through global task assignment and optimization, this approach enables cooperative robotic arms to perform accurate and efficient sorting, significantly improving overall system performance in real-world applications.

Classic sampling-based algorithms for robotic path planning include Rapidly-exploring Random Trees (RRT) and its optimized variant, RRT*. Reference [20] proposes Neural RRT* (NRRT*), which employs a non-uniform sampling distribution generated by a convolutional neural network (CNN). This model predicts the probability distribution of the optimal path on a map, guiding the sampling process and significantly improving the efficiency and memory usage of path planning. Reference [21] introduces a Bidirectional Inform-RRT* (BI-RRT*) algorithm, which enhances planning performance and reduces collision risk through informed sampling in both directions. Reference [22] presents the Quick-RRT algorithm, which expands upon RRT* by considering a broader range of parent nodes and applying the triangle inequality principle. This approach accelerates convergence and produces lower-cost paths. Reference [23] proposes the MOD-RRT* algorithm for navigation in unknown, dynamic environments. It generates high-quality initial paths and supports real-time replanning in response to environmental changes. Reference [24] further optimizes path costs through enhanced parent node selection, outperforming standard RRT algorithms. Reference [25] develops the HB-RRT algorithm using Halton sequences to mitigate the uneven sampling distribution seen in standard RRT. It also improves path quality through multi-level planning and cubic B-spline smoothing techniques. Reference [26] proposes a real-time mapping and shortest path planning algorithm for robotic arms using low-cost depth cameras. Based on the D* algorithm and grid maps, it generates Cartesian-space paths for the end-effector, with pose interpolation and smoothing applied to produce executable joint-space trajectories. Reference [27] introduces a sampling-based indefinite direction search algorithm for dual-arm cooperative manufacturing tasks, enabling adaptive responses in uncertain environments and enhancing task flexibility and cooperation. Reference [28] proposes a time-sampling-based operational space search strategy. By simplifying the map structure and determining the optimal local path segments within discrete time intervals, the method concatenates these segments to form collision-free paths. It demonstrates improved efficiency compared to traditional map search algorithms in multi-robot cooperative manufacturing systems.

With the rapid advancement of artificial intelligence, the learning-based approach has become a prominent area of research. Among its subfields, reinforcement learning [29] (RL) is widely recognized as a framework for formalizing sequential decision-making tasks. Unlike supervised or unsupervised learning, RL does not rely on labeled datasets; instead, agents learn optimal policies through trial-and-error interactions with dynamic environments, aiming to maximize the expected cumulative reward [30]. Traditional RL methods typically utilize Q-tables to represent policies, which are insufficient for high-dimensional action spaces such as those encountered in robotic arm control. Moreover, many deep reinforcement learning (DRL) models [31] are designed for discrete action spaces. In continuous domains, model-free algorithms have shown superior performance due to their strong decision-making capabilities and scalability to high-dimensional tasks [32]. Several DRL algorithms have demonstrated success in industrial automation, aerial robotics, and medical robotics. For example, Reference [33] proposes a multi-robot cooperative algorithm called MR-CDRL based on DRL. It uses end-to-end training on images generated from each robot’s relative viewpoint along with corresponding rewards, eliminating the need to predefine target positions or movement paths. This method resolves both resource contention and dynamic/static obstacle avoidance in real time. Reference [34] introduces the Twin Delayed Deep Deterministic Policy Gradient (TD3) algorithm, which employs dual Q-networks, delayed policy updates, and target policy smoothing to mitigate overestimation bias, rapid policy shifts, and instability—common limitations of the original DDPG framework. In the healthcare domain, Reference [35] employs the Proximal Policy Optimization (PPO) algorithm for robotic trajectory planning, achieving obstacle avoidance and task-specific trajectory generation. Reference [36] presents a trajectory planning method for robotic arms using the Multi-Agent Twin Delayed DDPG (MATD3) algorithm. It integrates a feedback controller and collision-avoidance mechanism to ensure safe operation in spatially constrained environments. For complex forestry tasks involving multi-log grasping, Reference [37] proposes a vision-servo-based multi-robot RL approach that enhances both grasping accuracy and efficiency. To improve success rates in grasping moving objects within unstructured environments, Reference [38] combines the MATD3 algorithm with a high-quality memory (H-memory) mechanism to form the MA-TD3H algorithm. This approach significantly outperforms conventional DRL methods in terms of success rate and time efficiency. Reference [39] introduces a multi-finger grasping method based on multimodal RL, utilizing fused multi-sensor data and dimensionality-reduced action spaces to achieve robust sim-to-real transfer. Experiments validate its superiority over baseline methods in real robotic systems. Lastly, Reference [40] proposes MO-MIX, a method for multi-objective multi-agent decision-making. Based on the Centralized Training and Distributed Execution (CTDE) framework, it inputs preference-weight vectors into distributed agents to condition local value estimations. A parallel hybrid network is used to compute joint value functions, and an exploration-guided strategy improves the diversity and coverage of Pareto-optimal solutions.

Traditional methods, with their explicit mathematical modeling and strong interpretability, have found widespread application in engineering control and path planning. However, such approaches still exhibit inherent limitations. Specifically: Artificial potential field methods can generate relatively smooth paths in real-time obstacle avoidance and online control scenarios, but they are prone to getting stuck in local minima, leading to path planning stagnation or unreachable goals. In unstructured environments like coal mines, this method lacks global search capabilities, making it difficult to obtain globally optimal paths in complex obstacle distributions or dynamic scenarios. Heuristic algorithms possess strong global search capabilities and can effectively avoid getting stuck in local optima. However, they require frequent replanning in dynamic or uncertain environments, resulting in insufficient real-time performance. They also struggle with trajectory optimization problems in continuous, high-dimensional spaces, such as those involving dual robotic arms. Sampling-based methods do not rely on precise environmental models, making them suitable for high-dimensional, complex, or unstructured scenarios. They can generate feasible paths in continuous spaces, aligning well with the characteristics of dual-robot arm systems. However, their sampling efficiency and path quality are highly dependent on the sampling distribution, leading to potential search imbalance. Real-time performance in dynamic environments also remains inadequate.

In summary, traditional algorithms demonstrate high stability and controllability in structured, deterministic tasks. However, their performance and adaptability are constrained in unstructured, dynamic, and high-dimensional continuous action spaces. For collaborative dual-arm grasping tasks in unstructured scenarios like coal mine conveyor belts, the system must possess both global search capabilities and meet real-time requirements.

Learning-based methods (DRL) demonstrate unique competitive advantages in continuous, high-dimensional action spaces: First, through policy gradient or value iteration mechanisms, agents can continuously refine strategies during interactions to meet real-time demands. Second, leveraging the nonlinear mapping capabilities of deep networks, the model can automatically extract spatio-temporal features from complex environments, reducing reliance on manual feature design. Third, the introduction of stochastic policies and noise perturbation mechanisms enables agents to perform global exploration in unknown spaces, avoiding local optima. Fourth, by integrating experience replay and multi-agent coordination mechanisms, the model maintains strong robustness and generalization capabilities even in dynamic or partially observable environments. Therefore, deep reinforcement learning methods demonstrate superior adaptability in dynamic environments, enabling trajectory optimization in continuous, high-dimensional action spaces while exhibiting robust generalization performance in unseen settings. This makes them an effective approach for tackling such complex tasks. To further validate the proposed method’s efficacy, subsequent sections will conduct experimental comparisons against mainstream deep reinforcement learning algorithms as a control group, highlighting the advantages and improvements achieved by this research.

However, learning-based methods still face technical bottlenecks: when handling continuous action space tasks for dual-arm cooperation, current mainstream reinforcement learning (RL) algorithms often suffer from policy estimation bias and insufficient exploration when confronting complex tasks involving coupled high-dimensional state and action spaces. To address these limitations, this study proposes the Multi-Agent Dual-Delay Deep Deterministic Policy Gradient (MATD3) algorithm as the core technical framework. This approach not only effectively mitigates policy estimation bias and insufficient exploration but also enables cooperative decision-making among multiple agents. It provides a robust technical foundation for dual-arm cooperative control in unstructured environments, demonstrating superior performance [41], specifically in the following aspects:

(1) Delayed update mechanism: Reduce the update frequency of the actor network to suppress the instability caused by strategy mutations.
(2) Target strategy smoothing: Mitigate Q-value overestimation issues by injecting action noise.

Based on this framework, three improvements are proposed:

① Design a reward function with multiple factors and constraints to accelerate the convergence of the robotic arm in the continuous motion space, thereby improving the real-time performance of the grasping trajectory for heterogeneous targets.
② Introduce a priority experience replay (PER) mechanism to accelerate strategy convergence to the optimal solution and enhance environmental dynamic adaptability through adaptive adjustment of sample priority weights and efficient reuse of experience data;
③ For slender objects, a sequence cooperative optimization strategy is developed to improve grasp-and-place stability and reliability.

This paper is organized as follows: Section 2 introduces the dual-arm constraint design, reward function design, and dual-arm task design. Section 3 provides a detailed introduction to the neural network design. Section 4 details the two experiments designed in this study and verifies the feasibility of the proposed method. Section 5 summarizes this study and concludes.

## 2. Problem Setting

### 2.1. Background and Setting

**Figure 1:**
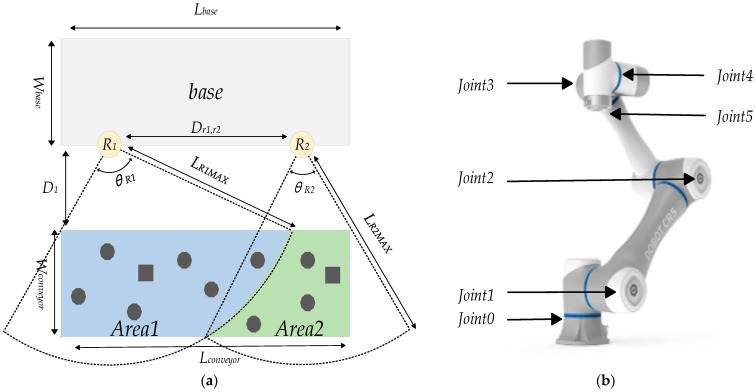
Defined declaration. (**a**) Top-down schematic of the dual-arm operational workspace; (**b**) definition of the robotic arm joints.

This study employs the PyBullet simulation environment, where all simulated objects are scaled at a 1:1 ratio to their real-world counterparts to ensure fidelity and transferability. The simulation task involves two CR5 robotic arms cooperatively removing foreign objects from a separate conveyor belt in a coal mine. The system dynamically adjusts its grasping strategy based on the geometry of each object, aiming to ensure safe, collision-free grasping and placement. A key prerequisite is that each robotic arm must be capable of rapidly and safely reaching any point within its defined reachable workspace. Figure 1 presents a top-down schematic of the dual-arm operational workspace and definition of the robotic arm joints.

In Figure 1, the polygons represent the coal flow, while the squares indicate the bounding box models of foreign objects within the workspace. It is important to note that these bounding box models are employed to ensure that randomly generated target points do not fall inside or intersect with coal blocks, thereby preventing invalid grasping attempts and enhancing the overall robustness of the planning strategy.

Assuming that i coal blocks are set up in the experiment, with the minimum corner point of the outer box being aabbMin = ($x_{\min}^{i}$, $y_{\min}^{i}$), and the maximum corner point being aabbMax = ($x_{\max}^{i}$, $y_{\max}^{i}$), the conditions for generating the target point are shown in Equation:

(1) $$\forall i\notin \left\{0,1,2,3\ldots n\right\},\left(x,y\right)\left[x_{\min}^{i}-\delta ,x_{\max}^{i}+\delta \right]\cdot \left[y_{\min}^{i}-\delta ,y_{\max}^{i}+\delta \right]$$

The target positions for both robotic arms must be reset at each training cycle in order to guarantee that the targets are diversified and accessible. This approach enables training the robotic arms to rapidly navigate to any cooperative point within the reachable domain without collision. Therefore, the significance of Equation (1) lies in preventing random target point generation from overlapping or conflicting with obstacles. This maintains geometric distribution rationality and ensures physical feasibility consistent with the real world. Among these, *δ* represents the buffer range, which is 0.03 m. *L_base_* = *L_conveyor_* = 1.5 m denotes the length of the robotic arm base and conveyor belt, *W_base_ = W_conveyor_* = 0.5 m respectively indicate the width of the base and conveyor belt, D_1_ = 0.4 m signifies the distance between the base and conveyor belt, *Dr*_1_,*r*_2_ = 1 m represents the distance between the dual-arm, *L_R_*_1_*_MAX_* = *L_R_*_2_*_MAX_* = 1.047 m represents the maximum reachable distance of the robotic arm. *Area* 1 and *Area* 2 represent the random target point generation regions for Robot 1 and Robot 2, respectively. These are divided into primary and secondary grasping regions. The practical significance of this classification lies in improving the efficiency of foreign objects grasping when the foreign objects content in coal is too high.

### 2.2. Dual-Arm System Constraint Settings

In order to guarantee safe, effective, and dependable task completion, limitations on the motion execution of twin robotic arms are necessary due to their structural complexity and the specific nature of the task. The following limitations are used in this work to direct the actions of the robotic arm:

#### 2.2.1. End-Effector Grasping Pose Constraints

To ensure the safety and stability of the si*x*-axis robotic arm’s end-effector across different motion configurations, specific joint angles near the end of the robotic arm are conditionally constrained. The objective is to guarantee that the end-effector consistently performs grasping actions in a top-down pose, minimizing the risk of collision and improving grasping reliability. The joint angle vector is defined as shown in Equation:

(2) $$R=\left[r_{0},r_{1},r_{2},r_{3},r_{4},r_{5}\right]$$

Here, *r*_0_ denotes the base rotation angle, *r*_1_ the shoulder joint angle, and so on. The shoulder and elbow joints primarily determine the approximate position of the robotic arm’s end-effector, while the wrist joints are responsible for fine-tuning its pose. In this study, the rotation angle of the elbow joint is used as a basis for segmented processing, through which the wrist joints *r*_3_ and *r*_4_ are adjusted accordingly to constrain the pose of the end-effector:

(3) $$r_{3}=\left\{\begin{array}{l} -\frac{\pi }{2}-r_{1}-r_{2},if\;r_{2}<0\\ -\frac{3\pi }{2}-r_{1}-r_{2},if\;r_{2}>0 \end{array}\right.$$

(4) $$r_{4}=\left\{\begin{array}{ll} -\frac{\pi }{2}, & \mathrm{if}\;r_{2}<0\\ \frac{\pi }{2}, & \mathrm{if}\;r_{2}>0 \end{array}\right.$$

$r_{1}$ denotes the rotational angle of the shoulder joint, describing the rotation of link 1 relative to the base; all circumferential directions in this study are counterclockwise. $r_{2}$ denotes the rotational angle of the elbow joint, describing the rotation angle of link 2 relative to link 1. $r_{3}$ denotes the rotational angle of wrist joint 1, describing the rotation angle of link 3 relative to link 2. $r_{4}$ denotes the rotation angle of wrist joint 2, describing the rotational angle of link 4 relative to link 3. Due to the multi-solution nature of robotic arm angle calculations, the above constraints ensure that the end-effector remains perpendicular to the ground during grasping operations, satisfying both obstacle avoidance and grasping requirements.

#### 2.2.2. Motion Amplitude Constraint

To ensure that joint movements remain within physical limits and to prevent instability during training caused by joint angle overflow, this study imposes constraints that restrict joint motions within predefined boundary intervals. Let $r_{t}\in R_{n}$ represent the robot joint angle at time t, $a_{t}\in R_{n}$ denote the action output by the intelligent agent at time *t*, and $a_{ct}$ denotes the constrained motion. This relationship is formally defined in Equation (5):

(5) $$a_{ct}=\min(\max(a_{t},a_{\min}),a_{\max})$$

where $a_{\max}$ and $a_{\min}$ denote the upper and lower limits of the action, respectively. Upon completing action $a_{t}$, it is first compared against the lower constraint limit $a_{\min}$. If the action $a_{t}$ exceeds the action $a_{\min}$, it is then compared against the upper constraint limit $a_{\max}$. The smaller of the two values is adopted as the action increment for the next update. Otherwise, the lower constraint limit is used as the action increment for the next update. After the action constraint is completed, the action is updated by a fixed step size, as shown in Equation (6):

(6) $$r_{t+1}=r_{t}+\Delta t\cdot a_{ct}$$

This study employs angle control mode for the robotic arm, expressed in radians. Directly applying the action output from the neural network to control the robotic arm would result in excessive movement amplitude, potentially introducing safety hazards. Therefore, after applying the action constraints, the action is updated by a fixed step size Δt. Where Δt is a real constant ranging from 0 to 1, designed to enable thorough exploration during the early training phase and refined utilization in the later phase. In this experiment, Δt is dynamically adjusted: when the number of training iterations is less than 300, Δt is set to 0.2; otherwise, it is set to 0.1.

#### 2.2.3. Cooperative Constraints in the Dual-Arm Cooperative Control Stage

In this study, to ensure the stability of the cooperative grasping and placing actions performed by the dual-arm, it is necessary to constrain the cooperation at different stages. The constraint is as shown in the Equation:

(7) $$\left\{\begin{array}{l} if\;up\;\;\;\Delta x、\Delta y\;=0\;\;z_{k}=z_{start1}+\frac{k}{N_{\mathrm{int}er}}\left(z_{end1}-z_{start1}\right)\\ if\;turn\;\;\Delta y、\Delta z=\;0\;\;x_{k}=x_{start}+\frac{k}{N_{\mathrm{int}er}}\left(x_{end}-x_{start}\right)\;\\ if\;down\;\;\Delta x、\Delta y\;=0\;\;\;z_{k}=z_{start2}+\frac{k}{N_{\mathrm{int}er}}\left(z_{end2}-z_{start2}\right) \end{array}\right.$$

In this equation, “*up*” refers to the upward phase of dual-arm foreign objects grasping. The objective of this phase is to achieve smooth upward grasping and movement of the foreign objects. The required motion trajectory is as follows: the *z*-axis cooperation increases with each time step, while the *x*, *y*-axis coordinates remain constant (i.e., Δx、Δy=0) Specifically, at the kth time step, the *z*-axis coordinate is $z_{k}$, $z_{start1}$ represents the *z*-coordinate during grasping,$z_{end1}$ denotes the endpoint of the *z*-axis during the upward phase, and $k=0,1\ldots N_{\mathrm{int}er}$ signifies the number of interpolation steps. In this study, all values are set to 100. The “turn” phase represents the transfer stage, aiming to move the objects from the conveyor belt to the placement area. The required trajectory is: the objects transitions from the x>0 area to the x<0 area as time steps increase. Specifically, at the *k*th time step, the *x*-coordinate is $x_{k}$, $x_{start}$ denotes the initial *x*-coordinate at the start of the transfer phase, and $x_{end}$ denotes the final *x*-coordinate at the end of the transfer phase. The “down” phase is the placement stage, aiming to smoothly lower the objects from top to bottom. The object’s trajectory requirement is that the *z*-axis decreases with increasing time steps while the *x* and *y* coordinates remain constant, i.e., Δx ,Δy =0.

### 2.3. Problem Statement

In this study, deep reinforcement learning methods are employed to address the cooperative grasping of foreign objects in coal mines using a single/dual robotic arm. Essentially, the interaction between the dual-arm and the environment is modeled as a Markov Decision Process (MDP), represented by the quintuple *M* = (*S*, *A*, *R*, *P*, *γ*), where: S denotes the state space, i.e., the set of all states; A denotes the action space, i.e., the set of all actions that the robotic arm can perform; *R*(*s*, *a*, *s*’) denotes the reward function, i.e., the immediate reward obtained by transitioning from state s to state s’ via action *a*; *P*(*s*’|*s*, *a*) denotes the state transition probability, i.e., the probability of the robotic arm reaching state s’ from state s after acting *a*; *γ* denotes the discount factor, used to balance short-term and long-term rewards, with *γ* ∈ [0, 1]. The ultimate goal of reinforcement learning is to maximize the expected value of cumulative rewards under the policy function. The expected total reward is defined as shown in Equation:

(8) $$J\left(\pi_{\theta }\right)=E_{\tau \sim \pi_{\theta }}\left[\sum_{t=0}^{\infty }\gamma^{t}r_{t}\right]$$

Among them, $\tau =(s_{0},a_{0},s_{1},a_{1}\ldots )$ represents a complete trajectory of the robotic arm, and rt is the instantaneous reward at time *t*. The learning process of the optimal strategy is to find a set of optimal parameters $\theta^{*}$ such that the cumulative reward obtained by the strategy in the environment is maximized:

(9) $$\theta^{*}=\arg\max J\left(\pi_{\theta }\right)$$

## 3. Method

### 3.1. Definition of State Space and Action Space

In deep reinforcement learning, the effective modeling of the state space and action space directly determines the learning efficiency of the agent. The network architecture takes the state space as input and outputs actions from the action space. These actions, after undergoing necessary constraint processing, are converted into control signals for the robotic arm, enabling the agent to continuously optimize its strategy during interactions to achieve task objectives. In the dual-arm cooperative task proposed by this research, the state space construction comprehensively considers the critical information required for task completion. It primarily consists of the following three components: (1) End-effector joint positions: To better describe the position of the robotic arm’s end-effector in the 3D coordinate system, this study incorporates the positions of each robotic arm joint in the 3D coordinate system as part of the state space. Let the joint position be denoted as *P*(x,y,z) representing coordinates values in the 3D coordinate system; joint number i = 0, 1, …, 5; robotic arm number j $j=\left\{1,2\right\}$. Then, the positions of the joints of the dual-arm in the 3D coordinate system can be expressed as: $P_{i}^{j}\in R^{3}$ (2) Displacement of the end-effector and target point along each axis: Let the position of the end-effector be $P_{e}^{j}\in R^{3}$ and the position of the target point be $P_{g}^{j}\in R^{3}$. Here, j denotes the robot arm number, $P_{e}$ denotes the end-effector position, and $P_{g}$ denotes the target point position. The displacement of the end-effector and target point along each axis can be expressed as $\Delta P^{j}=P_{g}^{j}-P_{e}^{j}$, where $\Delta P^{j}$ denotes the displacement between the end-effector and the target point. (3) Task Completion Flag: This flag indicates whether the current time step meets the task completion condition. It is a Boolean variable denoted as d, $d^{j}=True/False$. In summary, all state space components in this study are introduced. Therefore, the state space can be represented as Equation:

(10) $$S=\left[P_{0}^{j},\ldots P_{5}^{j},\Delta P^{j},d^{j}\right]$$

where S denotes the state space set. The dimensions of $P_{i}^{j}$, $\Delta P^{j}$ and $d^{j}$ are 36, 6, and 2, respectively. Thus, the state space in this study comprises 44 dimensions.

Regarding action space design, this paper employs a continuous action space. Each agent’s action vector comprises control inputs for six joint angles, specifically defined as:

(11) $$a_{t}=\Delta \theta_{i,j}$$

where $a_{t}$ denotes the action output by the network, Δθ represents the joint angle increment, *i* indicates the robotic arm joint number, and *j* denotes the robotic arm number. Consequently, the action space has a 12-dimensional structure. To ensure the physical feasibility of robotic arm movements, the angular range of each joint is subject to constraints, preventing unsafe incidents.

### 3.2. Control Strategies and Network Structures

In this study, the core control algorithm is based on the Multi-Agent Twin Delayed Deep Deterministic Policy Gradient (MATD3), which is employed to train single- and dual-arm robotic systems to perform designated tasks. Several enhancements were implemented to improve the algorithm’s performance. First, a multi-factor reward function was designed to leverage potential field gradient information, thereby driving the policy to converge efficiently in the continuous action space. Additionally, a priority experience replay (PER) mechanism was introduced to adaptively adjust sample priority weights and efficiently reuse experience data, accelerating convergence to the optimal policy and enhancing adaptability to dynamic environments. Finally, a sequential cooperative optimization strategy was developed for grasping slender objects, combining closed-loop control of the end-effector pose with a serialized operation workflow to ensure high reliability throughout the grasp-and-place process.

#### 3.2.1. Neural Network Structure

**Figure 2:**
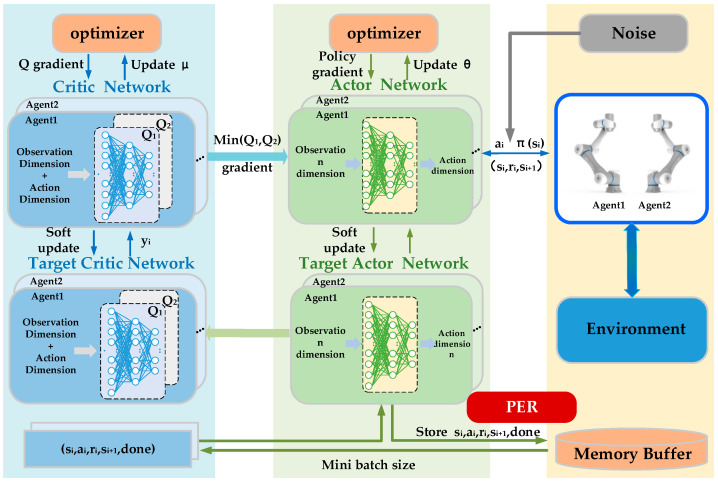
Algorithm’s network diagram.

MATD3 is a multi-agent reinforcement learning algorithm based on the Actor-Critic architecture, with its core being an extension of the single-agent TD3 (Twin Delayed DDPG) algorithm. TD3 effectively mitigates the value function overestimation issue present in its predecessor, DDPG (Deep Deterministic Policy Gradient), by introducing a dual Critic network and a delayed policy update mechanism. In MATD3, each agent maintains its independent Actor network (policy network) and dual Critic network (value function network), and adopts a centralized training and distributed execution (CTDE) paradigm. During the training phase, the Critic network can utilize global state information to perform more accurate joint value estimation to guide the optimization of each agent’s Actor policy; during the execution phase, it relies solely on the agent’s local observations to achieve distributed decision-making. The algorithm’s network diagram is shown in Figure 2.

Each agent’s neural network consists of three types of neural network structures: the policy network $\pi_{\theta }$, the critic networks *Q*1(*s*, *a*) and *Q*2(*s*, *a*), and the target network $\pi_{\theta }^{\prime }$. At each time step t, the MATD3 algorithm first obtains the current state $s_{t}$ from the environment and inputs this state into the corresponding policy network to generate the current action:

(12) $$a_{t}=\pi_{\theta }\left(s_{t}\right)+\varepsilon$$

Among them, ε is the exploration noise used to enhance exploration capabilities. After generating an action, the agent interacts with the environment, obtains immediate reward $r_{t}$, and observes the next state $s_{t+1}$. This process is repeated continuously, forming an interaction trajectory. To evaluate the effectiveness of the policy, the cumulative reward—referred to as the Return—is defined as the optimization objective:

(13) $$R_{t}=\sum_{i=t}^{T}\gamma^{i-t}r\left(s_{i},a_{i}\right)$$

Among these, *γ* ∈ (0, 1) is the discount factor, used to balance the relative importance of immediate rewards and future rewards. A smaller *γ* places greater emphasis on short-term returns, while a larger *γ* emphasizes long-term gains.

#### 3.2.2. Reward Function Design

In dual-arm cooperative grasping tasks, the design of the reward function is crucial, as it significantly affects the grasping efficiency and performance metrics of the robotic arms. To ensure that the dual robotic arms can accurately and safely complete the grasping task without collision, this paper designs a multi-factor reward function that comprehensively considers end-effector distance, collision factor, guidance metrics, and task completion degree. The reward function defined in this study is shown in Equation (14):

(14) $$R=R_{\mathrm{distance}}+R_{\mathrm{collision}}+R_{\mathrm{guidance}}+R_{\mathrm{done}}$$

Among them, $R_{\mathrm{distance}}$ is the distance from the end of the target point, $R_{\mathrm{collision}}$ is the collision factor, $R_{\mathrm{guidance}}$ is the guidance indicator, and $R_{\mathrm{done}}$ is the task completion rate.

(1) Distance component: $R_{\mathrm{dis}}=R_{base}+R_{add}$. Assuming that the current position of the end effector of the robotic arm is $P_{e}=\left(x_{e},y_{e},z_{e}\right)$, the target point position is $P_{g}=\left(x_{g},y_{g},z_{g}\right)$, and $\Delta P^{j}=P_{g}-P_{e}$ represents the deviation vector between the current position and the target positions:

(15) $$R_{\mathrm{distance}}=-\alpha \left\|P_{g}-P_{e}\right\|=-\alpha \cdot \sqrt{{\left(x_{g}-x_{e}\right)}^{2}+{\left(y_{g}-y_{e}\right)}^{2}+{\left(z_{g}-z_{e}\right)}^{2}}$$

where *α* is a custom parameter that adjusts the distance effect.

To encourage the robotic arm to accurately approach the target point, this paper designs a segmented reward mechanism: when $\Delta P^{j}$ is less than a certain threshold, the intelligent agent is given a certain positive reward $R_{\mathrm{add}}$:

(16) $$R_{add}=\left\{\begin{array}{l} \delta_{1},if\;d<0.2\\ 0,otherwise \end{array}\right.+\left\{\begin{array}{l} \delta_{2},if\;d<0.1\\ 0,otherwise \end{array}\right.+\left\{\begin{array}{l} \delta_{3},if\;d<0.01\\ 0,otherwise \end{array}\right.$$

In the formula, $\delta_{1},\delta_{2},\delta_{3}$ is a real number greater than 0, and b, $\delta_{1}<\delta_{2}<\delta_{3}$ are 0.1, 0.2, and 0.3, respectively, in this study.

(2) Collision part: In this study, collisions consist of three parts: self-collisions of the robotic arm, collisions between robotic arms, and collisions between the robotic arm and static obstacles. The reward function for this part is defined as shown in Equation (17):

(17) $$R_{\mathrm{collision}}=\left\{\begin{array}{l} R_{c},if\;collision\\ 0,otherwise \end{array}\right.$$

In the formula, $R_{c}$ is a negative real number, which $R_{c}$ is set to −1 in this experiment.

(3) Guidance reward component: In this study, a guidance reward mechanism was designed to enable the robotic arm to approach the target point quickly and efficiently. Assuming that the distance error at time t − 1 is $d_{t-1}$ and the distance error at the current time is $d_{t}$:

(18) $$R_{guidance}=\left\{\begin{array}{l} R_{g},if\;d_{t-1}>d_{t}\\ R_{g}^{\prime },if\;d_{t-1}<d_{t} \end{array}\right.$$

In the formula, $R_{g}\neq R_{g}^{\prime }$, $R_{g}>0,R_{g}^{\prime }<0$ and $\left|R_{g}\right|<\left|R_{g}^{\prime }\right|$. In this study, $R_{g}$ is 0.05 and $R_{g}^{\prime }$ is 0.1.

(4) Completion part: In this study, each training round is defined as having β time steps, and the number of times the robotic arm completes the task is defined as N. When the end-effector distance error is d<0.01, it is considered that the robotic arm has completed the task once, N + 1. When 50 consecutive completions are achieved within t time steps, it is considered that the task for this round is completed, and a reward $R_{done}$ is given to incentivize the robotic arm to perform the task better. In this study, $R_{done}$ is 10. The mathematical expression is shown in Equation (19):

(19) $$R_{done}=\left\{\begin{array}{l} R_{d},if\;N\geq 50\\ 0,if\;otherwise \end{array}\right.$$

#### 3.2.3. Priority Experience Replay

To enhance sample efficiency in multi-agent reinforcement learning, this study incorporates a prioritized experience replay (PER) mechanism. Unlike traditional experience replay that samples experiences uniformly at random, PER improves training efficiency by dynamically adjusting the sampling probability of each experience based on its temporal-difference (TD) error. Samples with larger TD errors—indicating higher learning potential—are given higher priority, allowing the agent to focus more on informative transitions and accelerate policy convergence.

Specifically, in each training round, each agent samples a uniform batch from the global experience replay buffer, where each experience includes the state, action, reward, next state, and termination flag of all agents. At the same time, in order to be compatible with the dual-critic architecture of TD3, the current *Q*-values of the two critic networks, *Q*_1_ and *Q*_2_, are calculated separately, along with the target Q-values, to obtain two TD errors:

(20) $$\sigma_{i}^{1}=\;Q_{1}(s,a)\;-\;Q^{t\arg et},\;\sigma_{i}^{2}=\;Q_{2}(s,a)\;-\;Q^{t\arg et}$$

Finally, the average of the two is used as an approximation of the TD error:

(21) $$\sigma_{i}\;=\;\frac{1}{2}(\sigma_{i}^{1}\;+\;\sigma_{i}^{2})$$

The sampling priority for each experience is set to the following:

(22) $$P_{i}={\left(\left|\sigma_{i}\right|+\varepsilon \right)}^{\alpha }$$

Based on this, the sampling probability $P_{i}$ is calculated. At the same time, in order to correct the bias caused by non-uniform sampling, importance sampling weights are introduced:

(23) $$\omega_{i}\;=\;{\left(\frac{i}{N_{\mathrm{buffer}}\cdot P(i)}\right)}^{\beta }$$

Among them, $N_{buffer}$ is the capacity of the experience replay buffer, and β controls the degree of compensation for sampling bias. To avoid violent bias caused by unstable estimates in the early stages of training, this paper sets β to linear growth:

(24) $$\beta_{t}\;=\;\beta_{0}\;+\;(\beta_{final}\;-\;\beta_{0})\cdot \frac{t}{T}$$

where *t* denotes the current training step and *T* denotes the total transition steps. This mechanism achieves a transition from weak PER to full PER, balancing exploration and convergence speed.

Under the priority experience replay mechanism, all agents share a unified experience replay buffer and sampling mechanism, but each updates independently based on its own Actor and Critic networks. Each agent uses shared samples but calculates actions using its own Actor network, trains its own Critic independently, and feeds TD error back to the experience replay buffer to update priorities. This design enables agents to share global experience while optimizing individually based on their own strategies, thereby improving sample utilization and strategy cooperation.

#### 3.2.4. Research on Sequence Cooperative Optimization Strategy

**Figure 3:**
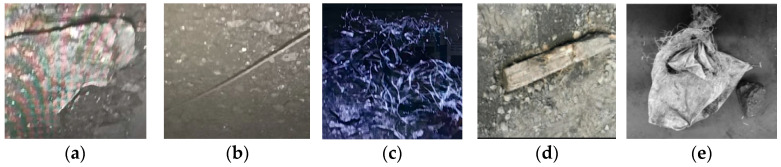
The classification of foreign objects in coal. (**a**) Ganguge; (**b**) Anchor rods; (**c**) Iron wire; (**d**) Woven bags; (**e**) Wood.

Common foreign objects encountered in coal transportation and processing primarily include gangue, anchor rods, metal wires, woven bags, and wood pieces. These foreign objects exhibit distinct characteristics and forms: (1) Coal gangue, as the primary impurity associated with coal mining, typically exhibits a particle size distribution ranging from 50 mm to 200 mm after mining and crushing processes, following specific statistical distribution patterns within this size range; (2) Metal wires and anchor rods are primarily concentrated in coal mining machinery operation zones. Due to factors such as underground production impacts and human activity disturbances, they appear in various states, including unfolded, coiled, or severed; (3) Woven bags and wood blocks predominantly originate from residual coal transport packaging materials and underground support materials. The incorporation of such foreign objects into coal is influenced by multiple factors including transportation processes and operational standardization, exhibiting distinct random characteristics in spatial distribution and inclusion probability. The classification of foreign objects in coal is shown in Figure 3.

Due to the heterogeneity and uncertainty of foreign objects in coal, this study focuses solely on grasping research for primary coal contaminants—namely gangue, anchor rods, and wood fragments—and temporarily excludes flexible foreign objects such as iron wire and woven bags. The targeted foreign objects fall into two categories: (1) Objects with concentrated centers of mass that can be grasped by a single arm, such as gangue and small-to-medium wood fragments; (2) Slender objects that cannot be grasped by a single arm, such as anchor rods and longer pieces of wood, which require dual-arm coordination for grasping. For the aforementioned foreign objects, this paper proposes a sequence cooperative optimization strategy based on the Minimum Bounding Box (MBB) model. Specifically, assuming that the MBB dimensions of the foreign objects in the three-dimensional world cooperative system are $\left[L_{x},L_{y},L_{z}\right]$, the sizes are first sorted as shown in Equation (25):

(25) $$\left[d_{1},d_{2},d_{3}\right]=sort\left(\left[L_{x},L_{y},L_{z}\right]\right),\;\;\mathrm{where}\;d_{1}>d_{2}>d_{3}$$

The foreign body classification criteria are defined in this study as follows in Equation (26):

(26) $$\mathrm{classification}=\left\{\begin{array}{ll} \mathrm{if}\;\frac{d_{1}}{d_{3}}<\tau_{1}, & \mathrm{Block}\text{-}\mathrm{Like}\\ \mathrm{if}\;\frac{d_{1}}{d_{2}}>\tau_{2}\;\mathrm{and}\;\frac{d_{2}}{d_{3}}<\tau_{3}, & \mathrm{Rod}\text{-}\mathrm{like} \end{array}\right.$$

Among them, $\tau_{1},\tau_{2},\tau_{3}$ are thresholds.

**Figure 4:**
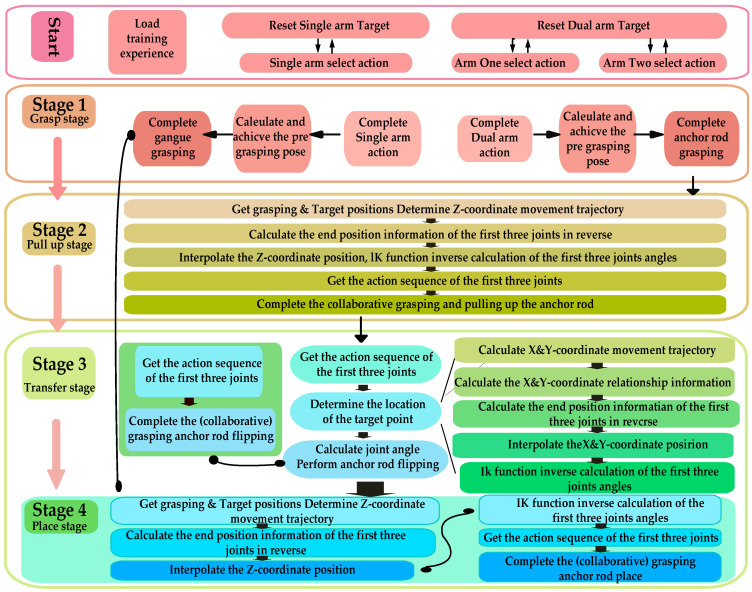
Algorithm Flowchart of the Sequence Cooperative Optimization Strategy.

The algorithm flowchart for the above sequence cooperative optimization strategy is shown in Figure 4.

Based on a sequence cooperative optimization strategy, this paper designed two experimental tasks for verification targeting typical foreign objects in coal mines. These tasks are single-arm independent grasping and dual-arm cooperative grasping, used to systematically evaluate the strategy’s actual execution effectiveness and operational performance under different grasping modes. As shown in Figure 3, the grasping task comprises five distinct phases:

(1) Model Experience Accumulation Stage: Periodic strategy evaluations are conducted during training, with experimental models saved at each time step to monitor performance changes and facilitate future utilization. The evaluation cycle spans 5000 time steps.
(2) Grasp Stage: The primary objectives are to drive the robotic arm based on the pre-trained model to reach the pre-grasping position; to obtain the position where the foreign object’s model is imported; and to calculate the robot arm’s pose for grasping the foreign objects based on the imported model position and execute the grasp.
(3) Pull Up Stage: To prevent large foreign objects from damaging the conveyor belt during movement and causing safety incidents, foreign objects placement requires three stages: ascent, transfer, and placement. The primary objective of the lifting phase is to achieve the following: stable vertical ascent of the robotic arm while gripping the foreign objects. Since only the *z*-axis coordinate changes during this process, while the x, *y*-axis coordinates remain constant, the end-effector’s coordinates and the robotic arm’s elbow joint coordinates are relatively fixed. Therefore, defining the end-effector’s coordinate changes equates to defining the elbow joint’s x,y-coordinate changes. This enables the precise motion trajectory of the robotic arm during the lifting phase to be obtained through interpolation and inverse kinematics. The specific mathematical derivation and calculation process will be presented in the subsequent part of this subsection.
(4) Transfer Stage: The objective of this phase is to transfer the foreign objects from the conveyor belt area (x > 0) to the safe placement area (x < 0). During this process, the x-coordinate decreases while the y- and z-coordinates remain constant. The end-of-arm coordinates can be derived mathematically from the end-of-arm coordinates. Similar to the ascent phase, the precise motion trajectory of the robotic arm can be obtained through interpolation and inverse kinematics calculations.
(5) Place Stage: The objective of this phase is to safely place the foreign objects. The *z*-axis coordinate continuously decreases, and the process is similar to the ascent phase, so it will not be repeated here.

The experimental tasks designed are as follows:

(1) Single-arm Independent Grasping Experiment Design

This experiment mainly focuses on exposed foreign objects with concentrated centers of gravity, which can be grasped independently by a single arm. The grasping algorithm is designed as follows (Algorithm 1):

**Algorithm 1:** Single-arm grasp
Load model experience Initialize model parameters For episodes in episodes_limit do      Reset environment      For step in step_limit do            for agent_id in 2 do                  if done_flags[agent_id] == True then                       agent[agent_id] hold action                  else                        agent[agent_id] choose action                  End if             End for             obs_next_n, rewards, done_n = step(a_n)            obs_n = obs_next_n            If all agents done then                    While not grip_done                            grip                            step simulation and delay                     End while                     Set move_action                     Execute action                     Break            End if      End for End for

(2) Experimental Design for Dual-arm Cooperative Grasping

This experiment mainly focuses on long, irregularly shaped foreign objects with an unbalanced center of gravity that are difficult for a single robotic arm to grasp stably. The specific algorithm design is shown below (Algorithm 2).

**Algorithm 2:** Dual-arm coordinated graspLoad model experience Initialize model parameters For episodes in episodes_limit do      Reset environment      For step in step_limit do            for agent_id in 2 do                  if done_flags[agent_id] == True then                      agent[agent_id] hold action                   else                        agent[agent_id] choose action                  End if             End for             obs_next_n, rewards, done_n = step(a_n)            obs_n = obs_next_n            If all agents done then                    Calculate the grasping position                     Execute anchor grasping sequence                         while not  gripper_anchor_done                              grip anchor                         step simulation and delay                         End while                 Calculate the upward sequence                         for agent_id in 2 do                              Execute anchor upward trajectory                        End for                Step simulation and delay                Calculate the turn sequence                         for agent_id in 2 do                              Execute anchor turn trajectory                       End for                  Step simulation and delay                  Calculate the down sequence                         for agent_id in 2 do                            Execute anchor down trajectory                      End for                 Break            End if      End for End for

In the experimental phase, to achieve stable placement operations, it is necessary to perform precise interpolation processing on the joints and end-effector pose of the robotic arm. The following sections will introduce the placement process in three stages: Pull up, Transfer, and Place. The interpolation strategies and implementation methods used are detailed as follows:

(3) Pull up stage

To achieve a smooth upward motion after grasping, this paper performs linear interpolation on the *z*-axis coordinates of the end-effector and combines inverse kinematics to solve for the corresponding joint angle trajectory. Since the trajectory changes of the end-effector primarily affect the pose changes of joint 3 during this process, this stage only performs inverse kinematics for joint 3 to obtain its angular trajectory as it changes with *z*-axis interpolation. Starting from grasping points $\left(x_{1},y_{1},z_{i}\right)$ and $\left(x_{2},y_{2},z_{i}\right)$, the end-effector position at step k is as follows:

(27) $$z_{i}^{k}\;=\;(1-\frac{k}{N})\cdot z_{start,i}\;+\frac{k}{N}\cdot z_{t\arg et,i},\;\;\;\;\;k=0,1,\ldots N$$

where $z_{i}\;=\;z_{start}$, $z_{t\arg et}\;=\;z_{start}\;+\;h$, *h* is the height of ascent, and to keep $x_{1},y_{1}$ and $x_{2},y_{2}$ unchanged, the target position for each step is as follows:

(28) $$P_{i}^{\left(k\right)}\;=\;\left[\begin{array}{l} x_{i}\\ y_{i}\\ z_{i}^{\left(k\right)}+\Delta z \end{array}\right]$$

Given an interpolation ratio of *N*, the interpolation ratio for step *i* is as follows:

(29) $$r_{i}\;=\;\frac{i}{N},\;\;i=\;0,1,2\ldots ,N$$

The end-point pose interpolation corresponding to each step is as follows:

(30) $$P_{1}^{\left(i\right)}=\;(x_{1}+\Delta x,y_{1}+\Delta y,(1-r_{i})z_{1}^{start}+r_{i}z_{1}^{t\arg et})$$

(31) $$P_{2}^{\left(i\right)}=\;(x_{2}+\Delta x,y_{2}+\Delta y,(1-r_{i})z_{2}^{start}+r_{i}z_{2}^{t\arg et})$$

**Figure 5:**
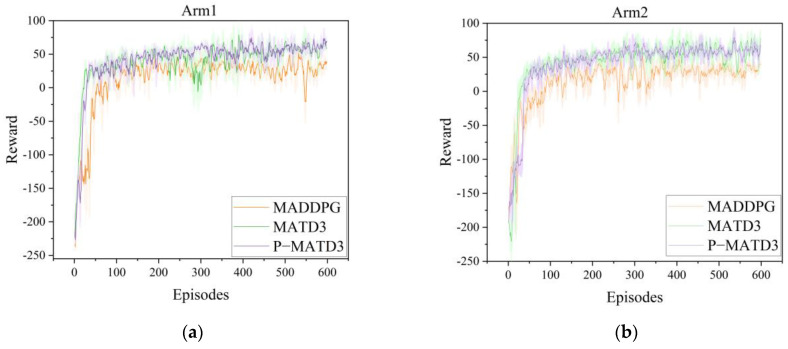
Different DRL algorithms training reward curve. (**a**) reward curve of robotic arm 1; (**b**) reward curve of robotic arm.

Among them, and are the differences between the end effector at the end of the ascending phase and joint3 on the *x*, *y* axis, as shown in Figure 5. Subsequently, the joint angle sequence of joint3 in the ascending phase is obtained by solving the inverse kinematics.

(32) $$q_{1}^{\left(i\right)}=IK_{1}(p_{1}^{\left(i\right)}),\;\;\;q_{2}^{\left(i\right)}=IK_{2}(p_{2}^{\left(i\right)})$$

(4) Transfer stage

To enable the dual-arm to perform coordinated turning movements after completing the grasping operation, linear interpolation of the *x*-axis coordinates is required. Unlike the ascending phase, when the *x*-axis coordinates change during the turning phase, the *y*-axis coordinates do not remain constant but instead form a composite trigonometric relationship with the base angle. Taking the starting points $\left(x_{1},y_{1},z_{1}\right)$ and $\left(x_{2},y_{2},z_{2}\right)$ as the initial positions, the interpolation of the end point *x*-axis is:

(33) $$x_{1}^{\left(i\right)}=(1-r_{i})\cdot x_{1}^{start}+r_{i}\cdot x_{1}^{\mathrm{target}}$$

(34) $$x_{2}^{\left(i\right)}=(1-r_{i})\cdot x_{2}^{start}+r_{i}\cdot x_{2}^{t\arg et}$$

The interpolation angles of the base joints are as follows:

(35) $$\theta_{1}^{\left(i\right)}=(1-r_{i})\cdot \theta_{1}^{\mathrm{start}}+r_{i}\cdot \theta_{1}^{t\arg et}$$

(36) $$\theta_{2}^{\left(i\right)}=(1-r_{i})\cdot \theta_{2}^{\mathrm{start}}+r_{i}\cdot \theta_{2}^{t\arg et}$$

Among them, $r_{i}$ is the difference ratio. Then, the joint position of joint 3 of robotic arm 1 is:

(37) $$x_{j1}^{\left(i\right)}=x_{1}^{\left(i\right)}-l_{4}\cdot \cos\theta_{1}^{\left(i\right)}-l_{3}\cdot \sin\theta_{1}^{\left(i\right)}$$

(38) $$y_{j1}^{\left(i\right)}=y_{1}^{\left(end\right)}-l_{4}\cdot \sin\theta_{1}^{\left(i\right)}-l_{3}\cdot \cos\theta_{1}^{\left(i\right)}$$

Correspondingly, the position of joint 3 of robotic arm 2 is:

(39) $$x_{j2}^{\left(i\right)}=x_{2}^{\left(i\right)}-l_{4}\cdot \cos\theta_{2}^{\left(i\right)}-l_{3}\cdot \sin\theta_{2}^{\left(i\right)}$$

(40) $$y_{j2}^{\left(i\right)}=y_{2}^{\left(end\right)}-l_{4}\cdot \sin\theta_{2}^{\left(i\right)}-l_{3}\cdot \cos\theta_{2}^{\left(i\right)}$$

In this case, $l_{4}$ and $l_{3}$ are the lengths of the mechanical arm links. Finally, the joint angle sequence for the turning phase can be obtained through inverse kinematics calculation:

(41) $$q_{1}^{\left(i\right)}=IK_{1}(x_{j1}^{\left(i\right)},y_{j1}^{\left(i\right)},z_{1})$$

(42) $$q_{2}^{\left(i\right)}=IK_{2}(x_{j2}^{\left(i\right)},y_{j2}^{\left(i\right)},z_{2})$$

(5) Place stage

The primary objective of this stage is to ensure that the *x* and *y* axis coordinates remain unchanged while the *z* axis coordinate is continuously interpolated to decrease. The interpolation method is similar to that described in Section 1 of this chapter, and further details will not be provided in this subsection.

## 4. Experimental Verification

(1) Comparison of different DRL algorithms in terms of training and capture effectiveness.

To validate the effectiveness of the proposed P-MATD3 algorithm in deep reinforcement learning training, the algorithm was trained alongside the MATD3 algorithm and MADDPG algorithm under the same configuration, with the same number of training rounds and the same number of steps per round. The training results reward function is shown in Figure 4. The network structure and hyperparameter settings are shown in Table 1 and Table 2. As shown in Figure 5, the P-MATD3 algorithm outperforms the other algorithms in terms of convergence speed and stability.

**Figure 6:**
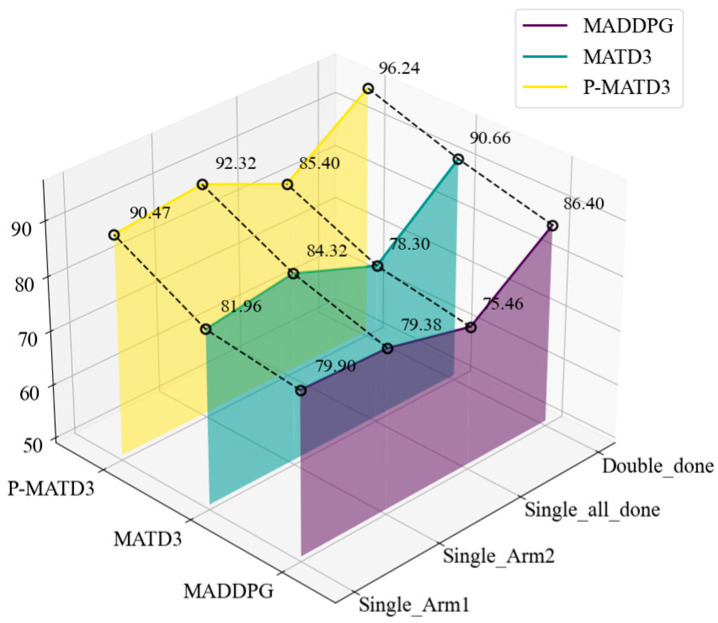
Robotic arm success rate under different tasks.

**Figure 7:**
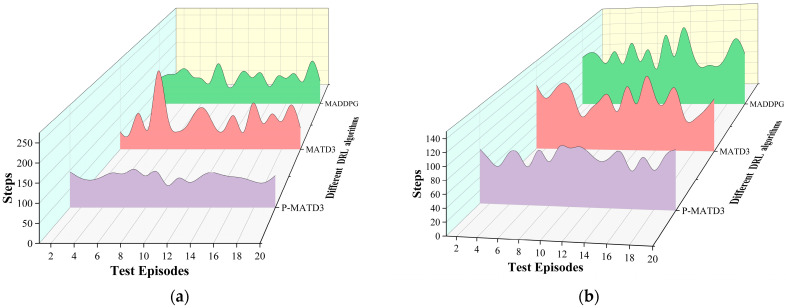
Robotic arm completion steps under different tasks: (**a**) single-arm completion steps; (**b**) dual-arm completion steps.

After training, the models generated by different algorithms were tested using a robotic arm: 50 tests were conducted for single-arm grasping of target objects and 50 tests for dual-arm cooperative grasping. The respective grasping success rates and completion steps are shown in Figure 6 and Figure 7.

As shown in Figure 6, when faced with single-arm foreign objects grasping, the P-MATD3 algorithm achieved a 7.1% improvement in grasping success rate compared to the MATD3 algorithm, and a 9.94% improvement compared to the MADDPG algorithm. When faced with dual-arm cooperative grasping, the P-MATD3 algorithm achieved a 5.58% improvement in success rate compared to the MATD3 algorithm, and a 9.84% improvement compared to the MADDPG algorithm. As shown in Figure 7, the P-MATD3 algorithm achieves shorter completion steps compared to the MATD3 and MADDPG algorithms when handling different tasks. Specifically, compared to the MATD3 and MADDPG algorithms, the P-MATD3 algorithm reduces the number of grasping steps by 11.44% and 12.77%, respectively, in single-arm grasping tasks. In the dual-arm task, reductions of 11.6% and 18.92% were achieved, respectively.

The preceding section demonstrated that the proposed method achieves high task success rates in interference-free environments. However, in real-world applications, sensor measurement errors, insufficient actuator precision, and environmental uncertainties all impact the decision-making process of intelligent agents. Therefore, it is necessary to further evaluate the algorithm’s stability and robustness under disturbed conditions. To this end, Gaussian noise was introduced to perturb the action space based on the trained model, and robustness experiments were conducted under these conditions to further validate the method’s stability. The validation results are shown in Table 3.

Experimental results indicate that when the Gaussian noise is 0.01, the algorithm’s performance remains largely consistent with that in a noise-free environment, exhibiting only slight fluctuations in success rate. When the amplitude of Gaussian noise increases to 0.05, the success rate decreases but still maintains a relatively high overall level, demonstrating that the method can sustain strong stability under moderate-intensity disturbances. When the Gaussian noise further increases to 0.1, performance degradation becomes more pronounced, yet a certain success rate is still maintained. In summary, this method demonstrates notable robustness under varying noise conditions. This validates the proposed method’s applicability in real-world complex environments.

**Figure 8:**
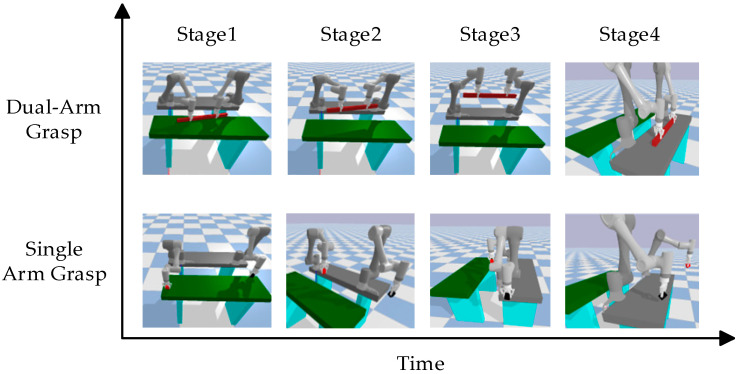
Grasping sequence diagram.

**Figure 9:**
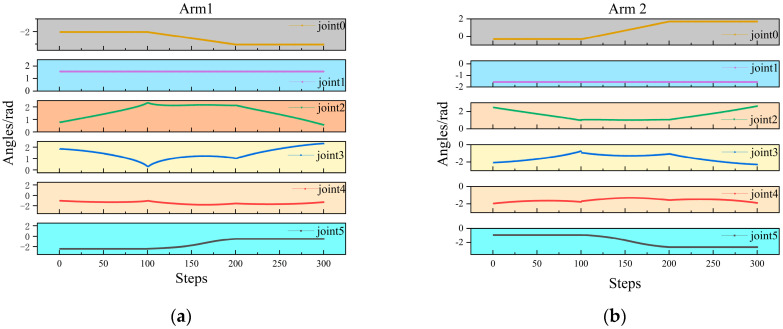
The angular velocity changes during the cooperative grasping process: (**a**) Arm 1 angular variation; (**b**) Arm2 angular variation.

**Figure 10:**
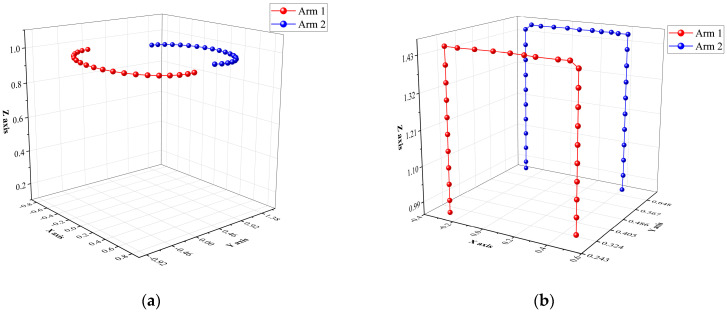
The end-effector trajectory variations: (**a**) single arm end-effector variation; (**b**) dual-arm end-effector variation.

Figure 8 shows the P-MATD3 algorithm in the PyBullet simulation environment using existing experience to grasp concentrated foreign objects and slender foreign objects with a robotic arm. Figure 9 and Figure 10 show the angular velocity changes during the cooperative grasping process and the changes in the end-effector trajectory variations of the dual-arm when performing different tasks.

(2) Analysis of module contributions

To validate the effectiveness of the proposed reward function and the applicability of priority experience replay, four experiments were carried out in this section to validate and analyze the contributions of individual modules., namely: the improved MATD3 algorithm (P-L-MATD3), the MATD3 algorithm with only the guided reward function (L-MATD3), the MATD3 algorithm with only priority experience replay (P-MATD3), and the original MATD3 algorithm. The experimental reward functions are shown in Figure 10.

**Figure 11:**
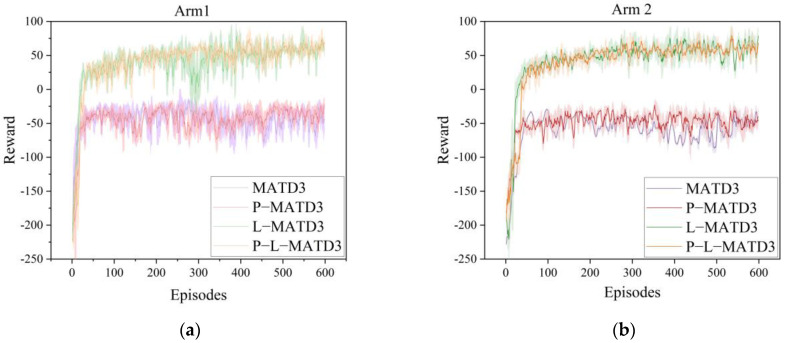
Different module training reward curve: (**a**) reward curve of robotic arm 1; (**b**) reward curve of robotic arm 2.

As shown in Figure 11, both P-L-MATD3 and L-MATD3 algorithms exhibit faster initial performance improvement; however, P-L-MATD3 demonstrates stronger stability in the later training stages and ultimately converges to superior performance. In contrast, P-MATD3 and MATD3 still show a certain degree of fluctuation during the later stages of training, indicating instability, and achieve relatively lower average rewards.

## 5. Conclusions and Future Work

### 5.1. Conclusions

This paper proposes a dual-arm cooperative grasping method for heterogeneous objects based on an improved MATD3 algorithm, tailored for unstructured environments. Its key innovations include the following:

(1) Designing a reward function that integrates multiple factors and constraints to accelerate convergence and optimize grasping trajectories;
(2) Introducing a priority experience replay mechanism into the algorithm, significantly enhancing sample utilization and policy learning efficiency;
(3) Proposing a sequential cooperative optimization strategy for slender heterogeneous objects to enhance stability during grasping and placement.

Experimental results demonstrate that the proposed method outperforms existing mainstream algorithms in grasping success rate, execution efficiency, and training stability. It exhibits robust performance under varying noise intensities, fully validating its application potential in complex coal mine environments.

### 5.2. Future Work and Challenges Ahead

**Figure 12:**
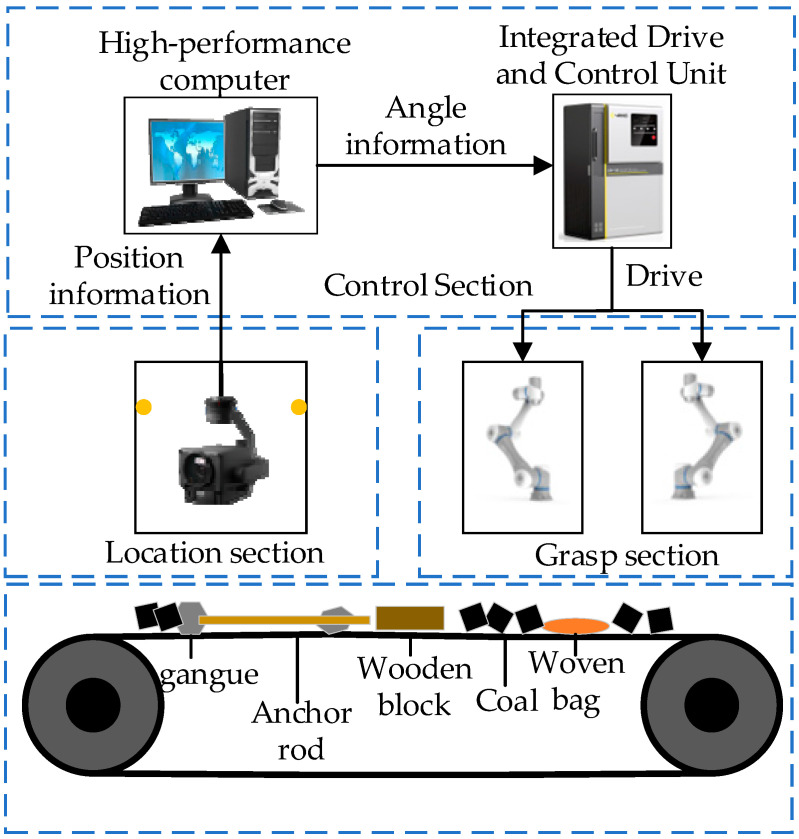
Test plan overall framework figure.

To validate the effectiveness and reliability of the algorithm in the real world, this paper proposes a test plan addressing the deployment challenge from simulation to reality. As illustrated in the figure, the plan consists of three components: the control section and the localization and grasping section. The control section comprises a high-performance computer and a robotic arm control mechanism; the localization and grasping section comprises a visual localization module and two CR5 robotic arms. The overall framework diagram is shown in Figure 12.

Below is a detailed introduction to the three major components:

(1) Positioning Component: This component utilizes deep learning-based image recognition technology to capture coal foreign object information at predetermined intervals along the conveyor belt. It records foreign object labels in real-time and transmits them to the control component. During operation, an industrial camera is positioned at the top center of the frame, with optimal capture frequency calibrated to enhance foreign object location detection.
(2) Control Module: This component encompasses model training, model loading, task strategy formulation, and trajectory planning. First, the enhanced deep reinforcement learning algorithm is trained and tested in a 1:1 simulation environment to achieve a specified accuracy threshold. During operation, the highest-performing model from training is loaded. It receives coal foreign object position queue information from the positioning module, then employs the trained model to formulate task strategies and plan trajectories, generating optimal grasping strategies in the simulation. Specifically, based on foreign object location and status data, it decides whether to deploy dual-arm grasping and assign tasks. Finally, it sends collision-free grasping trajectories to the control unit, directing the robotic arm to grasp foreign objects in coal like gangue, anchor rods, and wood blocks. Additionally, to comply with industrial standards and ensure experimental safety, an emergency stop button and a virtual fence will be installed on the robotic arm control unit.
(3) This section’s function is to execute the angular commands sent by the control unit. When a target foreign object can be grasped by a single robotic arm, the arm moves to the grasping position, performs the grasp, and safely deposits the object. When a target foreign object requires collaborative grasping, both robotic arms move to the pre-grasping positions. After grasping is completed, a sequential strategy is executed to ensure the foreign object is safely deposited.

This test plan comprehensively validates the task performance, robustness, and safety of the improved algorithm in real-world environments, providing a foundational framework for future research. Future work will incorporate Gaussian noise perturbations in real-world scenarios to further test the algorithm’s generalization capabilities.

Beyond this, numerous challenges remain for future research. For instance, the primary focus on kinematic planning for robotic arms idealizes the dynamics. However, real-world scenarios inevitably involve actuator issues such as sensor noise, latency, nonlinear friction, and motor saturation, presenting significant challenges. Simultaneously, dual-arm cooperation introduces significant safety risks (e.g., collisions and grasping failures) that simulations often fail to fully capture. To address this, a parallel perception architecture combining YOLO and GG-CNN enhances robustness and generalization, while fuzzy PID control enables precise manipulation of the end-effector.

## Figures and Tables

**Table 1:** Network structure parameter.

Layer	Actor Network	Activation	Critic Network	Activation
Input layer	22	Relu	22	Relu
Hidden laye r1	256	Relu	256	Relu
Hidden layer 2	128	Relu	128	Relu
Hidden layer 3	64	Relu	64	Relu
Output layer	6	Tanh	6	Tanh

**Table 2:** Hyperparameter settings.

Hyper-Parameter	Value	Hyper-Parameter	Value
Episodes_limit	600	Replay buffer size	2 × 10^4^
Step_limit	256	Exploration noise	0.5–0.2
Learning rate	5 × 10^−4^	Tau	0.0005
Batch size	256	Beta_start	0.4
Discount factor	0.99	Beta_frames	1 × 10^5^
epsilon	1 × 10^−6^	alpha	0.4

**Table 3:** Robustness test results under Gaussian noise.

The Value of Gaussian Noise	0.01		0.05		0.1	
Control mode	Single arm	Dual arm	Single arm	Dual arm	Single arm	Dual arm
Success rate	85.12%	95.17%	82.84%	93.42%	80.18%	90.14%

## Data Availability

Data are contained within the article.

## References

[1] Feng Z., Xue R., Yuan L., Yu Y., Ding N., Liu M., Gao B., Sun J., Zheng X., Wang G. (2025). Multi-agent Embodied AI: Advances and Future Directions. arXiv.

[2] Ma H., Zhang Y., Wang P., Cao X., Nie Z., Wei X., Zhou W., Zhang M. (2025). On the Academic Ideology of “Sorting the Gangue is Sorting the Images”. Coal Sci. Technol..

[3] He Z., Chu X., Liu C., Wu W. (2022). A Novel Model Predictive Artificial Potential Field Based Ship Motion Planning Method Considering COLREGs for Complex Encounter Scenarios. ISA Trans..

[4] Tang X., Zhou H., Xu T. (2024). Obstacle Avoidance Path Planning of 6-DOF Robotic Arm Based on Improved A* Algorithm and Artificial Potential Field Method. Robotica.

[5] Tian Y., Zhu X., Meng D., Wang X., Liang B. (2021). An Overall Configuration Planning Method of Continuum Hyper-Redundant Manipulators Based on Improved Artificial Potential Field Method. IEEE Robot. Autom. Lett..

[6] Xia X., Li T., Sang S., Cheng Y., Ma H., Zhang Q., Yang K. (2023). Path Planning for Obstacle Avoidance of Robot Arm Based on Improved Potential Field Method. Sensors.

[7] Lin H.-I., Shodiq M.A.F., Hsieh M.-F. (2025). Robot Path Planning Based on Three-Dimensional Artificial Potential Field. Eng. Appl. Artif. Intell..

[8] Fujimoto S., van Hoof H., Meger D. Addressing Function Approximation Error in Actor-Critic Methods. Proceedings of the 35th International Conference on Machine Learning, Stockholmsmässan.

[9] Völz A., Graichen K. An Optimization-Based Approach to Dual-Arm Motion Planning with Closed Kinematics. Proceedings of the 2018 IEEE/RSJ International Conference on Intelligent Robots and Systems.

[10] Hao L., Zhu X., Li T., Sang S., Cheng Y., Ma H., Zhang Q., Yang K. (2022). An Improved Path Planning Algorithm Based on Artificial Potential Field and Primal-Dual Neural Network for Surgical Robot. Comput. Methods Programs Biomed..

[11] Lee S., Jeong E., Oh M., Oh C. (2019). Driving Aggressiveness Management Policy to Enhance the Performance of Mixed Traffic Conditions in Automated Driving Environments. Transp. Res. Part A Policy Pract..

[12] Wang W., Zhu M., Wang X., He S., He J., Xu Z. (2018). An Improved Artificial Potential Field Method of Trajectory Planning and Obstacle Avoidance for Redundant Manipulators. Int. J. Adv. Robot. Syst..

[13] Lu X., Welleck S., West P., Jiang L., Kasai J., Khashabi D., Le Bras R., Qin L., Yu Y., Zellers R. NeuroLogic A*esque Decoding: Constrained Text Generation with Lookahead Heuristics. Proceedings of the 2022 Conference of the North American Chapter of the Association for Computational Linguistics: Human Language Technologies.

[14] Yu X., Chen W.-N., Gu T., Yuan H., Zhang H., Zhang J. (2019). ACO-A*: Ant Colony Optimization Plus A* for 3-D Traveling in Environments With Dense Obstacles. IEEE Trans. Evol. Comput..

[15] Luo M., Hou X., Yang J. (2020). Surface Optimal Path Planning Using an Extended Dijkstra Algorithm. IEEE Access.

[16] Nazarahari M., Khanmirza E., Doostie S. (2018). Multi-objective Multi-robot Path Planning in Continuous Environment Using an Enhanced Genetic Algorithm. Expert Syst. Appl..

[17] Liu J., Wang H., Li X., Chen K., Li C. (2022). Robotic Arm Trajectory Optimization Based on Multiverse Algorithm. Math. Biosci. Eng..

[18] Ekrem O., Aksoy B. (2023). Trajectory Planning for a 6-Axis Robotic Arm with Particle Swarm Optimization Algorithm. Eng. Appl. Artif. Intell..

[19] Ma H., Wei X., Wang P., Zhang Y., Cao X., Zhou W. (2022). Multi-Arm Global Cooperative Coal Gangue Sorting Method Based on Improved Hungarian Algorithm. Sensors.

[20] Wang J., Chi W., Li C., Wang C., Meng M.Q.-H. (2020). Neural RRT*: Learning-Based Optimal Path Planning. IEEE Trans. Autom. Sci. Eng..

[21] Fan H., Li J., Zhang Y., Zhou J. (2024). BI-RRT*: An Improved Path Planning Algorithm for Secure and Trustworthy Mobile Robots Systems. Heliyon.

[22] Jeong I.-B., Lee S.-J., Kim J.-H. (2019). Quick-RRT*: Triangular Inequality-Based Implementation of RRT* with Improved Initial Solution and Convergence Rate. Expert Syst. Appl..

[23] Qi J., Yang H., Sun H. (2021). MOD-RRT*: A Sampling-Based Algorithm for Robot Path Planning in Dynamic Environment. IEEE Trans. Ind. Electron..

[24] Liao B., Wan F., Hua Y., Ma R., Zhu S., Qing X. (2021). F-RRT*: An Improved Path Planning Algorithm with Improved Initial Solution and Convergence Rate. Expert Syst. Appl..

[25] Zhong H., Cong M., Wang M., Du Y., Liu D. (2024). HB-RRT: A Path Planning Algorithm for Mobile Robots Using Halton Sequence-Based Rapidly-Exploring Random Tree. Eng. Appl. Artif. Intell..

[26] Wang L., Qi Y., Li W., Liu M., Zhang Z. (2023). Dynamic Parallel Mapping and Trajectory Planning of Robot Arm in Unknown Environment. IEEE Sens. J..

[27] Su C., Xu J. (2022). A Sampling-Based Unfixed Orientation Search Method for Dual Manipulator Cooperative Manufacturing. Sensors.

[28] Su C., Xu J. (2022). A Novel Non-Collision Path Planning Strategy for Multi-Manipulator Cooperative Manufacturing Systems. Int. J. Adv. Manuf. Technol..

[29] Matsuo Y., LeCun Y., Sahani M., Precup D., Silver D., Sugiyama M., Uchibe E., Morimoto J. (2022). Deep Learning, Reinforcement Learning, and World Models. Neural Netw..

[30] Du W., Ding S. (2021). A Survey on Multi-Agent Deep Reinforcement Learning: From the Perspective of Challenges and Applications. Artif. Intell. Rev..

[31] Munikoti S., Agarwal D., Das L., Halappanavar M., Natarajan B. (2024). Challenges and Opportunities in Deep Reinforcement Learning with Graph Neural Networks: A Comprehensive Review of Algorithms and Applications. IEEE Trans. Neural Netw. Learn. Syst..

[32] Cui Y., Xu Z., Zhong L., Xu P., Shen Y., Tang Q. (2025). A Task-Adaptive Deep Reinforcement Learning Framework for Dual-Arm Robot Manipulation. IEEE Trans. Autom. Sci. Eng..

[33] Wang D., Deng H., Pan Z. (2020). MRCDRL: Multi-Robot Coordination with Deep Reinforcement Learning. Neurocomputing.

[34] Haarnoja T., Zhou A., Abbeel P., Levine S. Soft Actor-Critic: Off-Policy Maximum Entropy Deep Reinforcement Learning with a Stochastic Actor. Proceedings of the 35th International Conference on Machine Learning.

[35] Prianto E., Kim M., Park J.-H., Bae J.-H., Kim J.-S. (2020). Path Planning for Multi-Arm Manipulators Using Deep Reinforcement Learning: Soft Actor–Critic with Hindsight Experience Replay. Sensors.

[36] Tang W., Cheng C., Ai H., Chen L. (2022). Dual-Arm Robot Trajectory Planning Based on Deep Reinforcement Learning under Complex Environment. Micromachines.

[37] Jiang D., Cai Z., Peng H., Wu Z. (2021). Coordinated Control Based on Reinforcement Learning for Dual-Arm Continuum Manipulators in Space Capture Missions. J. Aerosp. Eng..

[38] Huang Y., Liu D., Liu Z., Wang K., Wang Q., Tan J. (2024). A Novel Robotic Grasping Method for Moving Objects Based on Multi-Agent Deep Reinforcement Learning. Robot. Comput.-Integr. Manuf..

[39] Liang H., Cong L., Hendrich N., Li S., Sun F., Zhang J. (2022). Multifingered Grasping Based on Multimodal Reinforcement Learning. IEEE Robot. Autom. Lett..

[40] Hu T., Luo B., Yang C., Huang T. (2023). MO-MIX: Multi-Objective Multi-Agent Cooperative Decision-Making with Deep Reinforcement Learning. IEEE Trans. Pattern Anal. Mach. Intell..

[41] Ficuciello F., Migliozzi A., Zaccara D., Villani L., Siciliano B. (2019). Vision-based Grasp Learning of an Anthropomorphic Hand-arm System in a Synergy-based Control Framework. Sci. Robot..

